# Complementary and Alternative Therapies for Liver Diseases

**DOI:** 10.1155/2013/656192

**Published:** 2013-12-25

**Authors:** Yong-Song Guan, Mohammad Ahmad Al-Shatouri, Qing He

**Affiliations:** ^1^Department of Oncology and Radiology, West China Hospital of Sichuan University, Chengdu, Sichuan 610041, China; ^2^Suez Canal Medical School, Suez Canal University, Ismailia 41522, Egypt

Alternative and complementary therapies for liver disease are undergoing a rapid development in the fields of theoretical research, laboratory study, and clinical practice. However, their safety, efficacy, and mechanisms of action remain insufficiently understood and even controversial. And incidences of many types of liver disease are currently rising. So there is an urgent need for the clarification of their safety, efficacy, quality assurance, and reproducibility. 

That a plant heals better than a tablet sounds marvelous, especially for such an organ as the liver which is complicated in both structure and function. Definite proof is exceptionally valuable for the establishment of methods to cure a group of disease with worldwide prevalence. The accumulated evidence on researches in liver disease will set a foundation for these cheaper, more holistic, more person-centered, or customizing therapies. 

This special issue contains the research papers focusing on current general interest in these therapies, including everyday life utensils and nourishment. A number of materials used in these therapies are subject to our study, such as pollen, bee honey, plant extracts, concoctions, officinal TCM pellets, and herbal injections. Convincible evidence is presented from *ex vivo* and *in vivo* studies, as well as clinical trials for the support of their hepatoprotective, antiviral, and anticancer effects in the basis of pharmacological sciences, molecular biology, and molecular medicine.

*Yong-Song Guan* *Yong-Song Guan* *Mohammad Ahmad Al-Shatouri* *Mohammad Ahmad Al-Shatouri* *Qing He* *Qing He*

